# Ankle Strategies for Step-Aside Movement during Straight Walking

**DOI:** 10.3390/jcm12165215

**Published:** 2023-08-10

**Authors:** Lingchao Xie, Sanghyun Cho

**Affiliations:** Department of Physical Therapy, Yonsei University, Wonju 26493, Republic of Korea; stanforia09@gmail.com

**Keywords:** fall risk, walking mechanics, neural control mechanisms, ankle strategies, gait stability

## Abstract

The step-aside movement, also known as the dodging step, is a common maneuver for avoiding obstacles while walking. However, differences in neural control mechanisms and ankle strategies compared to straight walking can pose a risk of falling. This study aimed to examine the differences in tibialis anterior (TA), peroneus longus (PL), and soleus (SOL) muscle contractions, foot center of pressure (CoP) displacement, and ground reaction force (GRF) generation between step-aside movement and straight walking to understand the mechanism behind step-aside movement during walking. Twenty healthy young male participants performed straight walking and step-aside movements at comfortable walking speeds. The participants’ muscle contractions, CoP displacement, and GRF were measured. The results show significant greater bilateral ankle muscle contractions during the push and loading phases of step-aside movement than during straight walking. Moreover, the CoP displacement, GRF generation mechanism, and timing differed from those observed during straight walking. These findings provide valuable insights for rehabilitation professionals in the development of clinical decisions for populations at a risk of falls and lacking gait stability.

## 1. Introduction

Step-aside movement (dodging step) during walking is a common maneuver to avoid obstacles; however, it is associated with fall risk owing to differences in neural control mechanisms and ankle strategies compared to straight walking. In our previous study, the mechanisms of ankle muscle contraction, foot center of pressure (CoP) displacement, and ground reaction force (GRF) generation in step-aside movements during standing were explored, excluding the influence of walking [1]. The current study aimed to extend previous findings by studying these mechanisms during straight walking, offering potential avenues for understanding obstacle avoidance.

Straight walking is generated by a central pattern-generating network in the spinal cord and controlled by supraspinal signals. In walking disturbances, corticospinal and interspinal neurons are activated to reduce their impact. Changes in walking direction are achieved through supraspinal control signals [2,3]. The corticospinal tract controls precision motor tasks, such as obstacle avoidance, and requires tight interactions between voluntary and automatic control of body equilibrium [4,5]. The mediolateral ankle strategy compensates by shifting the CoP relative to the center of mass, which improves coordination and balance [6,7]. This strategy has been extensively studied in various contexts, such as side-stepping [8], step turning [9], and gait termination [10]. Yet, the underlying mechanism of the step-aside movement remains largely unknown. In related side-stepping movements (widening the standing base), the peroneal longus (PL), tibialis anterior (TA), and soleus (SOL) are primarily activated to shift the complementary CoP, ensuring mediolateral gait stability [6]. However, in daily life, the step-aside movement is more commonly employed for obstacle avoidance than the side-stepping movement; the success rate of side-stepping for obstacle avoidance is relatively low [11]. Understanding the step-aside mechanism during walking is crucial for enhancing the targeted training for the elderly or individuals with limited mobility to reduce the risk of falls during obstacle avoidance. Our previous study explored the mechanisms of the left push and right loading phases in step-aside movements during standing. This study builds upon previous findings by examining the differences in ankle muscle contraction, foot CoP displacement, and GRF generation between step-aside movements and straight walking, thereby determining the ankle joint strategy and control mechanism in step-aside movements during straight walking.

Building on a previous study, which demonstrated the crucial role of the PL in the push and loading phases of step-aside movement and the substantial contribution of the TA to toe clearance in the push phase [1], we hypothesized that:

1. The PL would exhibit the greatest contraction during the left push phase of the step-aside movement, and all three ankle muscles would show increased contractions in the right loading phase compared to straight walking.
2. The TA would have the most significant contraction to achieve left-toe clearance.

Ankle muscle contractions displace CoP [12], and the step-aside movement requires a greater mediolateral travel distance than straight walking; however, the forward movement remains unchanged, as demonstrated by Novacheck et al. [13]. Therefore, we hypothesized that:

3. Mediolateral foot CoP (CoPx) displacement would increase medially during the left push phase of the step-aside movement. However, anteroposterior foot CoP (CoPy) displacement would be the same as in straight walking during both the left push and right loading phases [14].
4. The left forefoot GRF (F-GRF) and right heel GRF (H-GRF) would increase stepwise with rightward movement.

This understanding of the step-aside mechanism could serve as a basis for clinical decision-making prior to rehabilitation interventions and for the development of targeted rehabilitation protocols, reducing the risk of falls and improving stability during obstacle avoidance in elderly individuals and patients. Additionally, this knowledge could provide a theoretical foundation for our future research and development targeting obstacle avoidance training.

## 2. Methods

### 2.1. Participants

Twenty healthy young male participants (23.30 ± 2.15 year; 176 ± 5.12 cm; 70.83 ± 6.09 kg; mean ± standard deviation) were recruited, with a foot size of 260–270 mm owing to the size of the foot insole sensors. Individuals with balance issues, pes planus, chronic ankle instability [15], or previous major lower extremity problems were excluded.

All participants provided written informed consent before each test session. The Yonsei University Mirae Institutional Review Board approved the experimental study protocol (approval no.1041849-202207-BM-127-02). This study was conducted in accordance with the Declaration of Helsinki. Written informed consent was obtained for publication of the images.

### 2.2. Experimental Procedure

Experimental trials were conducted in a 36 m straight corridor, and the participants walked in two conditions at normal walking speed (1.15 ± 0.13 m/s) between three parallel lines of yellow pinstripe tape on the corridor floor at shoulder-width distances as per walking guidelines [16,17]. The participants walked straight between the left and middle guidelines; ten steps were statistically analyzed.

**Figure 1:**
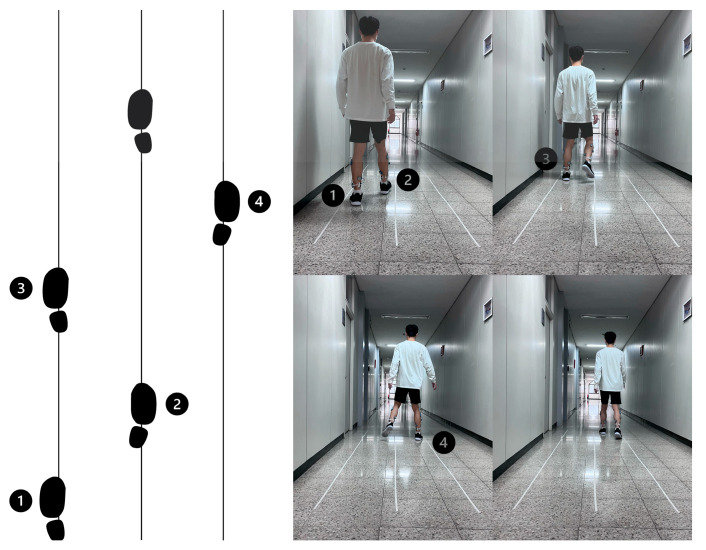
The sequence of “step-aside to the right” during straight walking. The numbers indicate the self-verbal cue.

Afterward, the participants walked straight within the left and middle guidelines again, but this time, they were instructed to step aside to the right to walk between the middle and right pinstripe tapes at a self-selected time. Since a change in direction must be planned in the previous step [9], participants dictated verbal cues of “1, 2, 3” to themselves while they made step-aside movements. The specific foot placement of the step-aside movement and timing of the self-verbal cues are shown in Figure 1. Ten step-aside movements were statistically analyzed.

When individuals perform movements in a novel or unfamiliar physical environment, the cerebellum registers errors in motor commands and implements co-contractions to counteract external disturbances [18,19]. However, with repeated practice, appropriate motor commands are learned and errors are reduced. Babadi et al. demonstrated that co-contraction significantly decreased, and motor learning significantly increased in the third of three repetitive movement sessions [20]. Therefore, in this study, the participants performed three sessions for both walking conditions, and only the third session was recorded and analyzed. The data from the first and last steps of each condition were not included in the statistical analysis.

### 2.3. Data Collection

#### 2.3.1. Electromyography

Noraxon Ultium (Noraxon Inc., Scottsdale, AZ, USA) wireless surface electromyography (EMG) sensors were used to record bilateral activities of the PL, TA, and SOL muscles. As recommended by SENIAM [21], the electrodes were placed 20 mm apart with respect to the muscle fiber to be measured. The skin was prepared by shaving and cleaning with an alcohol swab before electrode attachment. The raw signals were recorded and processed using MR3.18^®^ software (Noraxon Inc., Scottsdale, AZ, USA). EMG data were recorded at a sampling frequency of 2000 Hz, high-pass filtered at 20 Hz, rectified, and low-pass filtered at 50 Hz, as described by Rankin et al. [22]. For amplitude normalization, the field-rectified EMG data were divided by their average values during straight walking, and the EMG percentage data relative to normal walking was used for statistical analysis.

#### 2.3.2. Force and Center of Pressure

F-GRF, H-GRF, and foot CoP displacement data were collected using knitted shoes (Natso Knit Jogging Shoes, Keon Jong, Republic of Korea) with a Noraxon Ultium Insole (Noraxon Inc., Scottsdale, AZ, USA). The Noraxon Ultium Insole utilizes 8 sensors positioned in critical areas of the foot, and the collected sensor data undergo processing using interpolation techniques to accurately approximate (hysteresis < 5%, error < 3%) the pressure distribution across the entire foot [23]. During the development phase of the Noraxon Ultium insole, Noraxon reported that they conducted extensive testing to compare the body weight-normalized ground reaction force measured with a pressure platform and force-instrumented (Gaitway) treadmill [24]. In the present study, an increase in CoP displacement represented a shift to the right (medial side of the left foot and lateral side of the right foot) and anterior sides. A decrease in CoP displacement indicated the opposite. CoP movement was analyzed in the mediolateral and anteroposterior directions. F-GRF and H-GRF data were normalized according to the body weight of each participant by the Noraxon insole automatically. All data traces were cut at the heel strike, with the time normalized to 200 data points per gait cycle to obtain a high data resolution.

### 2.4. Statistical Analysis

A power analysis using G-Power software 3.1.9.4 (Franz Faul, University of Kiel, Germany) was performed to compute the minimum sample size requirement. The sample size was determined to be 15 according to an effect size (eta squared, η^2^ = 0.8) and power (1 − β = 0.81) on the difference in EMG_Lt.PL between the step-aside movement and straight walking. The data utilized for statistical analysis comprised the grand means, calculated as the average of all experimental movements across all participants.

The present study tested the differences between the conditions in EMG muscle activity, CoP displacement, and GRF by statistically examining the entire time series using one-dimensional statistical parametric mapping (SPM1d) in Python 3.8 (www.python.org, accessed on 8 August 2023), using the open-source software package SPM1d 0.4.8 [25] (www.spm1d.org, accessed on 8 August 2023).

#### 2.4.1. Region of Interest

Because we recorded the entire gait cycle, a region of interest (ROI), which is a portion or subdomain of the 1D measurement domain [26], was set to analyze the data within a specific time region. Because the H-GRF and F-GRF gradually decreased and increased, respectively, during the step-aside movement, the left foot ROI was set between 19% and 67% of the gait cycle to confirm differences in the left push phase between the step-aside movement and straight walking. The intersection of the H-GRF and F-GRF trajectories during a single support period for all participants was identified, and the average was set as the starting point. The mean position at which the left F-GRF reached zero was set as the endpoint (end of the left stance phase). The right foot ROI was set between 0% and 22.5% of the gait cycle to identify differences in the right loading phase between the two conditions. The endpoint was set at the average position, where the right F-GRF and H-GRF trajectories intersected.

A two-tailed paired *t*-test was used to investigate the differences in ipsilateral/bilateral EMG activities, CoP displacement, and GRF between step-aside movements and straight walking. SPM{t} was created by calculating the conventional univariate t-statistic at each muscle activation point [27]. Hotelling’s T^2^ test, the vector field equivalent to the two-sample *t*-test [28,29] was used to check the magnitude and time period of the ipsilateral/bilateral EMG activation differences. The magnitude at each time point was calculated using the scalar output statistic, SPM{T2}. SPM{t} and SPM{T2} indicated the magnitude of the differences. Based on random field theory, we calculated the critical threshold at which only α% (5%) of the smooth random curves were expected to traverse to test the null hypothesis (H0) [30], which is a valid approach for 1D data [31,32].

#### 2.4.2. Multiple Linear Regression

Multiple linear regression analysis was conducted for the statistically significant dependent variables identified by the SPM analysis to determine the correlation between the variables in the CoP and GRF models. Moreover, we estimated how the causal relationships among the variables differed between step-aside movements and straight walking. Only data within the selected ROI (left: 19–67%, right: 0–22.5% gait cycle) were used for statistical analysis.

The CoP model comprised multiple linear regression analysis with CoPx or CoPy displacement as the dependent variable and TA, PL, and SOL EMG amplitudes as the independent variables. For the GRF model, F-GRF or H-GRF were the dependent variables, and CoPx, CoPy displacement, and TA, PL, and SOL EMG amplitudes were independent variables. Multiple linear regressions were conducted using JASP (version 0.16.2.0; JASP Team, 2022).

When comparing ankle muscle EMG activations, CoP displacements, and GRFs between bilateral step-aside movements and straight walking, most critical SPM{t} thresholds were exceeded. Therefore, the null hypothesis was rejected.

## 3. Results

The two-tailed paired *t*-test revealed significant increases in EMG activation of all three ankle muscles during the left push and right loading phases of the step-aside movement compared to straight walking.

**Figure 2:**
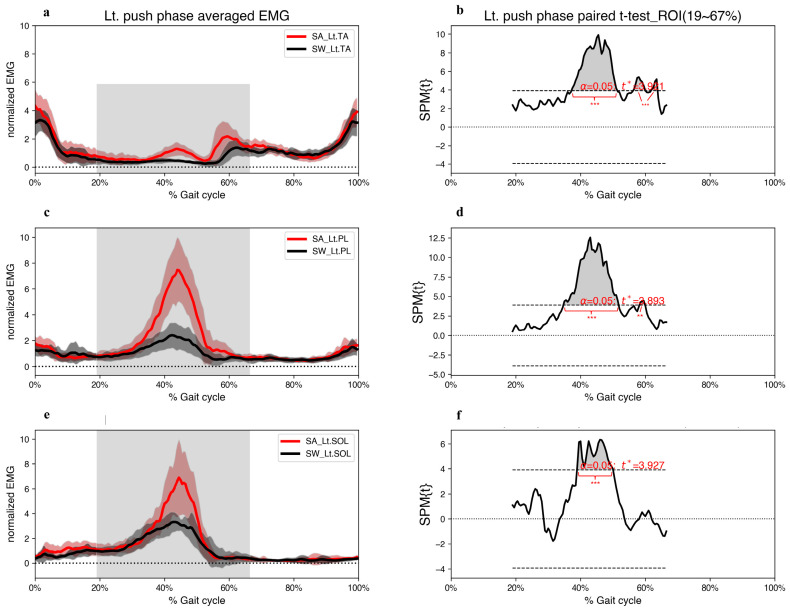
(**a**,**c**,**e**) Grand mean patterns for normalized TA, PL, and SOL EMG amplitudes of the step-aside to right movement (red) and the straight walking (black) in the left push phase. The grey bars indicate the ROI. (**b**,**d**,**f**) SPM{t} results depict the differences in muscle activities between the two conditions. The critical threshold t* (wide dashes) exceeded during (**b**) 37.2–51.4%, 56.2–59.9%, and 61.7–63.8%, (**d**) 34.7–51.8%, and 58.2–59.9%, (**f**) 38.9–50.0% of the gait cycle. *p* values < 0.05 (*), <0.01 (**), and <0.001 (***) indicate significant differences between SA and SW. TA; tibialis anterior, PL; peroneus longus, SOL; soleus, SA; step-aside movement, SW; straight walking, ROI; region of interest.

During the left push phase, the EMG amplitudes of the left PL (EMG_Lt.PL) showed the largest magnitude of difference above the critical threshold compared to those of the other ankle muscles (Figure 2). Similar to the EMG amplitudes of the left TA (EMG_Lt.TA), a major region of the SPM{t} trajectory (suprathreshold cluster-shaded area) exceeded the critical threshold, indicating statistically significant differences at the end of the left push phase (Figure 2b,d). Additionally, SPM analysis revealed significantly greater activation of all three left ankle muscles during the step-aside movement than that of straight walking during 38.9–50.0% of the gait cycles (Figure 2).

**Figure 3:**
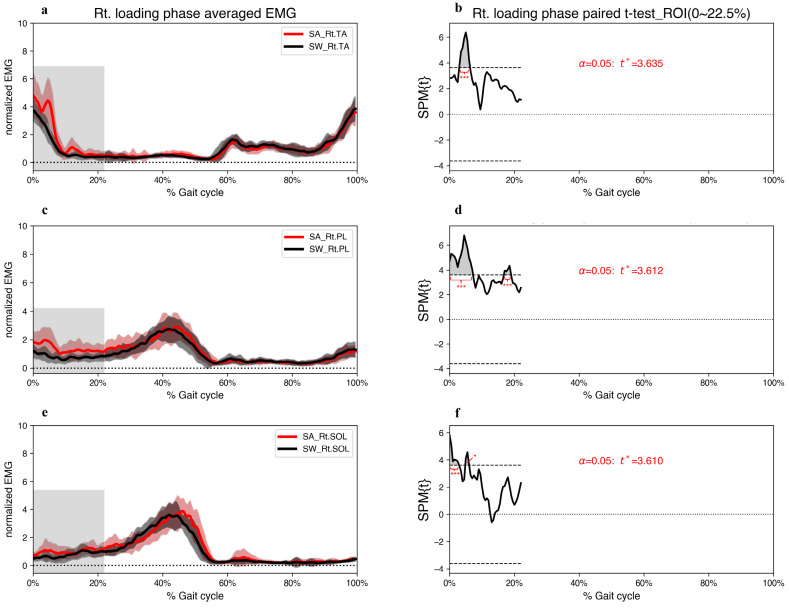
(**a**,**c**,**e**) Grand mean patterns for normalized TA, PL, and SOL EMG amplitudes of the step-aside to right movement (red) and straight walking (black) in the right loading phase. The grey bars indicate the ROI. (**b**,**d**,**f**) SPM{t} results depict the differences in muscle activities between the two conditions. The critical threshold t* (wide dashes) exceeded during (**b**) 3.0–6.3%, (**d**) 0–7.2% and 16.7–19.0%, (**f**) 0–2.9%, and 4.8–6.0% of the gait cycle. *p* values < 0.05 (*), and <0.001 (***) indicate significant differences between SA and SW. TA; tibialis anterior, PL; peroneus longus, SOL; soleus, SA; step-aside movement, SW; straight walking, ROI; region of interest.

At the beginning of the right loading phase, the EMG amplitudes of the right TA (EMG_Rt.TA) contracted the most among the three ankle muscles (Figure 3). However, the SPM results indicate significantly greater activation of all three ankle muscles in the right foot during the step-aside movement than that during straight walking during 4.8–6.0% of the gait cycles (Figure 3). Additionally, the SPM analysis revealed that the EMG_Rt.PL muscle had the largest proportion of major regions (grey area in Figure 3d) throughout the gait cycles, and only the EMG_Rt.PL muscle showed statistically significant differences during 16.7–19.0% of the gait cycles (Figure 3d).

**Figure 4:**
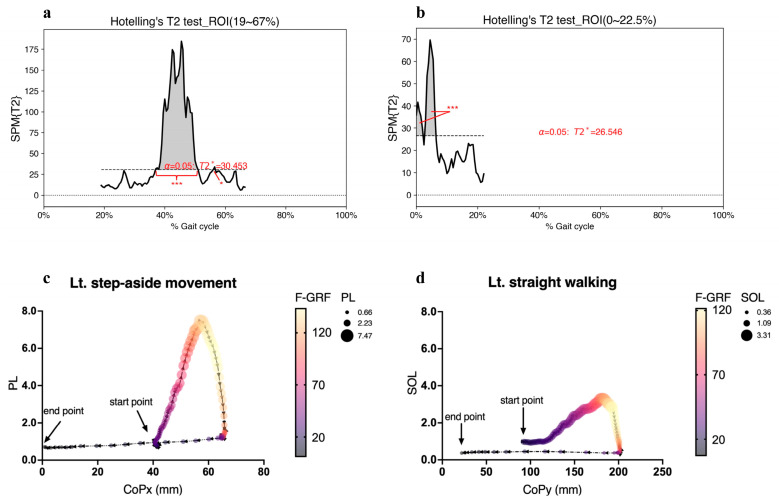
(**a**,**b**) SPM{T2} results (vector-field Hotelling’s T2 test statistic trajectory) depict the differences in muscle activations between step-aside to right movement and straight walking in the (**a**) left push phase, and (**b**) right loading phase. The critical threshold T2* (wide dashes) exceeded during (**a**) 36.9–51.1% and 56.0–56.8% of the left push phase, and (**b**) 0–2.3% and 2.7–6.5% of the gait cycle. *p* values < 0.05 (*), and <0.001 (***) indicate significant differences between SA and SW. SA; step-aside movement, SW; straight walking, ROI; region of interest. (**c**,**d**) Multiple linear regression for the left GRF model (F-GRF) with the two most relevant independent variables. Increasing CoPx and CoPy indicate movement towards the right (medial) and anterior, respectively. The *y*-axis indicates normalized EMG amplitudes. Each data point represented by a continuous circle conveys three pieces of information: the magnitude of muscle contraction, position of the center of pressure (CoP), and magnitude of the ground reaction force (F-GRF). The size of the circular representation, in conjunction with its corresponding value on the *y*-axis, represents the muscle contraction, while the value indicates the CoP position on the *x*-axis. The hue of the representation serves as a visual representation of the F-GRF. The arrows indicate the CoP trajectory from heel contact (start point) to toe-off (endpoint). F-GRF values are in % BW.

Hotelling’s T2 test showed a significantly different coordination pattern of the ankle muscles in the step-aside movement from that in straight walking during the left push and right loading phases. The magnitude of the differences and major regions of the T2 trajectory exceeding the critical threshold are shown in Figure 4.

**Figure 5:**
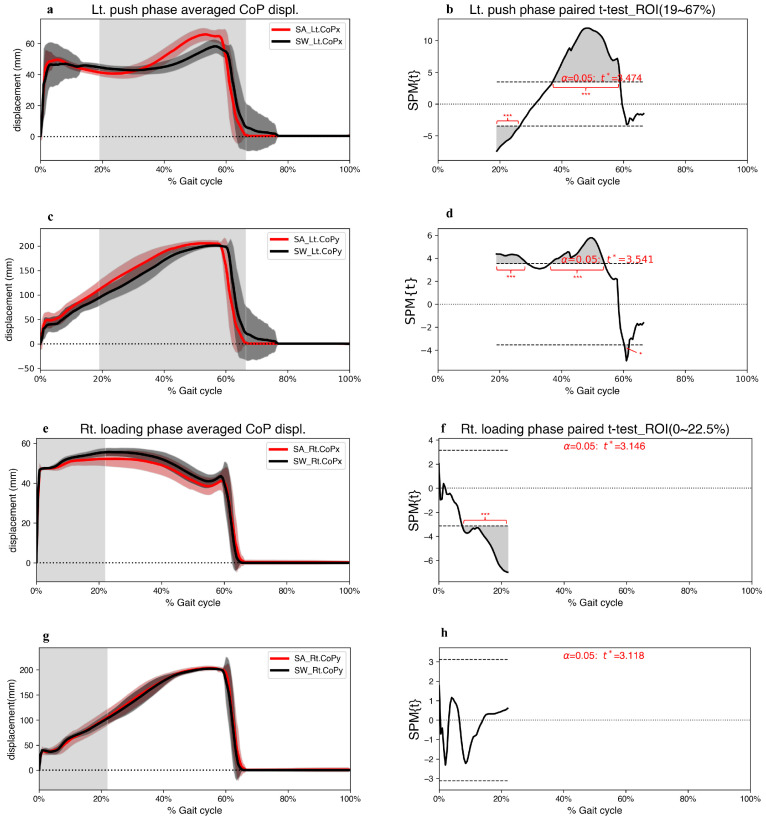
(**a**,**c**,**e**,**g**) Grand mean patterns for CoP displacement of the step-aside to the right movement (red) and the straight walking (black) in the left push and right loading phase. The grey bars indicate the ROI. (**b**,**d**,**f**,**h**) SPM{t} results depict the differences in CoP displacement between the two conditions. The critical threshold t* (wide dashes) exceeded during (**b**) 19.0–26.5% and 37.0–58.9%, (**d**) 19.0–28.9%, 36.3–53.9%, and 60.3–61.8%, (**f**) 7.5–22.0% of the gait cycle. *p* values < 0.05 (*), and <0.001 (***) indicate significant differences between SA and SW. CoP, center of pressure; SA, step-aside movement; SW, straight walking; ROI, region of interest.

In the left push phase, the two-tailed paired *t*-test indicated a significant decrease in foot CoPx displacement at 19.0–26.5%, and a significant increase at 37.0–58.9% of the gait cycle (Figure 5b). Furthermore, foot CoPy displacement significantly increased from 19.0–28.9% and 36.3–53.9%, with a significant decrease at 60.3–61.8% of the gait cycle. However, the right-foot CoPx displacement significantly decreased only during 7–22.0% of the gait cycle compared to straight walking (Figure 5f).

**Figure 6:**
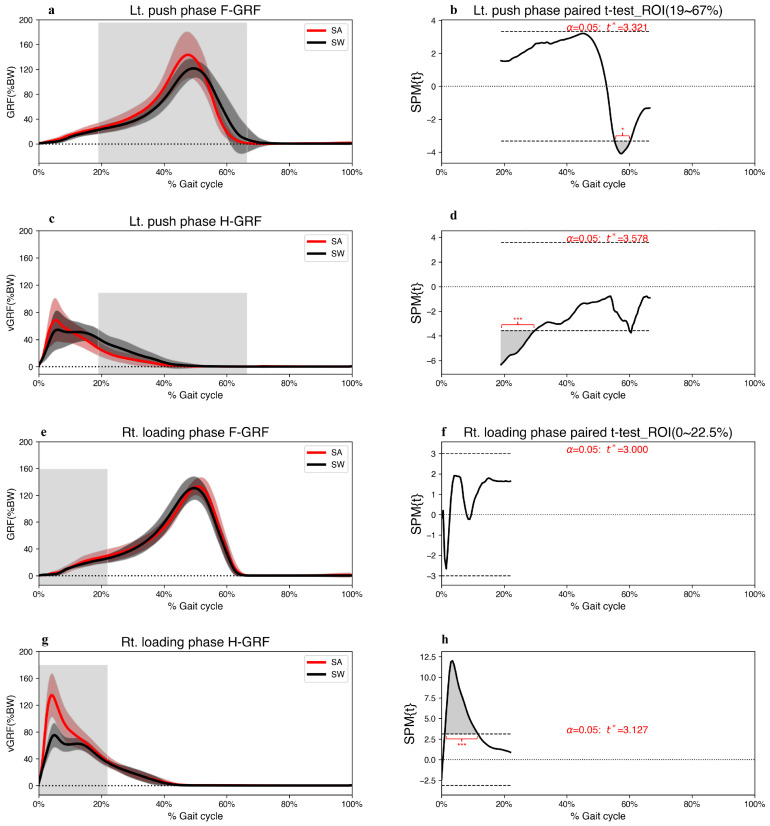
(**a**,**c**,**e**,**g**) Grand mean patterns for F-GRF and H-GRF of the step-aside to the right movement (red) and straight walking (black) in the left push and right loading phase. The grey bars indicate the ROI. (**b**,**d**,**f**,**h**) SPM{t} results depict the differences in F-GRF between the two conditions. The critical threshold t* (wide dashes) exceeded during (**b**) 55.3–60.2%, (**d**) 19.0–29.7%, (**h**) 1.0–11.6% of the gait cycle. *p* values <0.05 (*), and <0.001 (***) indicate significant differences between SA and SW. F-GRF, forefoot ground reaction force; H-GRF, heel ground reaction force; SA, step-aside movement; SW, straight walking; ROI, region of interest.

The SPM{t} trajectory of the left-foot F-GRF and H-GRF crossed the critical threshold downward, representing a significant decrease compared to straight walking (Figure 6b,d). Additionally, the SPM analysis revealed that during the right loading phase of the step-aside movement, a major region of the SPM{t} trajectory of the right-foot H-GRF exceeded the critical threshold, signaling a remarkable and significant increase in step-aside movement (Figure 6h).

### Regression

SPM analysis revealed statistically significant differences between step-aside movement and straight walking for TA, PL, and SOL contractions, CoPx and CoPy displacements, and F-GRF in the left push phase. Multiple linear regression analyses were performed for the left CoP (left CoPx, left CoPy) and left GRF (left F-GRF) models. PL contraction dominated the mediolateral ankle strategy in step-aside movements (Table 1), which aligns with our previous findings [1]. F-GRF generation was strongly correlated with independent variables in the left push phase for both movements, indicating that the combination of ankle muscle contraction and CoP displacement significantly influenced F-GRF generation. Figure 4c,d illustrates the changes in ankle muscle contraction (most correlated) and F-GRF during CoP displacement in the left push phase for both movements. Figure 4c shows that PL contraction and F-GRF increased as CoPx moved medially.

Similarly, Figure 4d shows a simultaneous increase in SOL contraction and F-GRF during the forward displacement of CoPy. However, comparing Figure 4c,d shows that PL contraction was larger than SOL contraction, and the F-GRF generated during the step-aside movement was more significant than that generated during straight walking, indicating that the step-aside movement induced more PL-induced F-GRF generation than SOL-induced F-GRF generation during straight walking.

Furthermore, statistically significant differences were found between step-aside movement and straight walking regarding TA, PL, and SOL contractions, and H-GRF in the right loading phase. Consequently, we performed multiple linear regressions exclusively on the right GRF model (right H-GRF), as shown in Table 1.

## 4. Discussion

Our results reveal significantly higher bilateral ankle muscle activation during step-aside movement than during straight walking in the selected period of the left push phase, particularly in the left PL muscle, as previously reported [1]. The results support our hypothesis that the CoP trajectories in the left push phase within the selected ROI would significantly differ. Significant differences were found in the CoPx and CoPy trajectories. In contrast, during the right loading phase, CoPx displacement significantly decreased but CoPy displacement changed minimally. Additionally, regression analysis confirmed a significant decline in left-foot H-GRF and improvement in right-foot H-GRF.

SPM analysis verified hypothesis 1. During the step-aside movement, the left ankle muscles begin to contract sequentially after the onset of the terminal stance period (34% of the gait cycle). The EMG_Lt. PL and EMG_Lt.SOL peaked at approximately 45% of the gait cycle. Hotelling’s T2 test identified a peak during this period, indicating significantly greater ankle muscle contraction than during straight walking.

The SPM analysis also verified our second hypothesis, with greater muscle contractions in EMG_Lt.TA and EMG_Lt.PL than those during straight walking in the pre-swing period (58.2–59.9% of the gait cycle) to complete toe clearance.

Contrary to our hypothesis 3, participants shifted the body weight earlier to the left to complete the step-aside movement during 19.0–26.5% of the gait cycle (Figure 5). A previous study revealed that during anticipatory postural adjustments (APA), humans move their foot CoP in the opposite direction to generate initial forward propulsive force [33]. In the present study, step-aside movement can be considered a new direction concerning forward straight walking. Similar to APA, shifting the left-foot CoP in the opposite direction (lateral, left) benefits muscle contractions (PL) to produce a right-side propulsive force. We previously observed a similar phenomenon in step-aside movements during standing [1]. Commencing at 37.0% of the gait cycle, the left-foot CoP moved swiftly towards the medial side and covered a greater distance in a shorter time in the medial and anterior directions, which could be because the next step was taken in a different direction. Therefore, along with the effects of the APA-like mechanism, the left-foot CoP moved further to provide sufficient propulsive force. Foot CoP displacement in the step-aside movement ended significantly sooner than in straight walking (Figure 5d). This shorter stance phase can be attributed to the need to move longer distances in a shorter time, as demonstrated by a study of the running mechanism [13]. The participants in our study took the next step longer than the normal stride length. Thus, the left-foot stance phase ended early to ensure sufficient time for the right foot to land in the appropriate position. This was supported by our EMG results, which indicate significantly greater muscle activation of the EMG_Lt.TA and EMG_Lt.PL (*p* < 0.001) than those during straight walking in the pre-swing period (Figure 2).

F-GRF increased during the left push period (Figure 6a); however, the SPM analysis failed to detect a statistical increase owing to a high standard deviation. Nonetheless, all the left ankle muscles and left foot CoP displacements displayed statistically significant differences during 38.9–50.0% of the gait cycle in the step-aside movement. The F-GRF significantly decreased during the left pre-swing period. Regression analysis revealed a strong correlation between PL muscle activation and foot CoPx displacement, generating propulsive force (Figure 4c). Unlike straight walking, where the SOL and CoPy displacements dominated F-GRF generation, PL contraction contributed significantly during the step-aside movement. However, the resulting F-GRFs showed only relatively small statistically significant differences (*p* < 0.01) over a short period. (Figure 4c,d and Figure 6). Although the PL contributed more than the SOL, the SOL and foot CoPy displacements were significantly greater than those during straight walking (Figure 2e,f). Therefore, we inferred that F-GRF generation during the left push phase of the step-aside movement was based on SOL contraction and foot CoPy displacement (Figure 4d), along with an additional contribution from the PL-induced foot CoPx displacement in the direction of change.

During the right loading phase, similar muscle activation patterns to our previous study were observed, supporting the hypothesis of increased ankle stability during initial foot contact [1]. All three ankle muscles showed significant increases (*p* < 0.001) during the right initial contact (double support period), with the EMG_Rt.PL exhibiting a more extended period of significant difference. Moreover, the right PL muscle also showed significant activation during 16.5–19.3% of the gait cycle (loading response, single-support period), indicating its role in maintaining ankle joint stability during both double and single support in the step-aside movement.

PL muscle activation increases with a greater mediolateral inclination of the walking surface to prevent excessive ankle inversion and resist ankle inversion during the stance phase of gait [34]. In the current study, the medial side of the right foot touched the ground before the lateral side in the step-aside movement, creating a mediolateral incline between the sole and ground. This resulted in PL activation, which developed an eversion torque to maintain stability as the body weight shifted to the front right.

SPM analysis revealed a significant decrease in right-foot CoPx displacement, indicating a more medially positioned right-foot CoP during initial contact in the step-aside movement. Furthermore, significant differences were found in the H-GRF during the right loading phase, indicating an increase in stride length compared to straight walking and a heightened H-GRF upon loading [35]. However, all EMG variables in the right GRF model of step-aside movement were excluded because of multicollinearity, making it difficult to establish a causal relationship between the right foot GRF and EMG or CoP displacement.

## 5. Limitation

To minimize data inaccuracies due to gender differences, we recruited only young men for the experiment, therefore, the results may vary for other populations. The movements performed by participants in this study were pre-planned, which may not fully replicate the spontaneous step-aside movements applied in real-life obstacle avoidance. Due to the experiment’s constraints in a relatively long corridor, data collection was limited to portable EMG sensors and insoles, allowing for only the acquisition of EMG, CoP, and vertical GRF data for analysis. As a result, joint moment, kinematic data, and medial/lateral, anterior/posterior GRF measurements were not obtained. Future studies should address these aspects for a more comprehensive understanding of the biomechanical factors involved in the analyzed tasks.

## 6. Conclusions

During step-aside movement, ankle muscle contractions were higher in push/loading phases compared to straight walking, indicating a greater risk of stumbling for individuals with weakened ankle muscles. Adequate preparation time is crucial for safe step-aside movements, and hasty actions to avoid obstacles may lead to collisions and falls. In the push phase, the F-GRF (forefoot vGRF) is generated through SOL and PL muscle contraction, enabling directional changes. In the loading phase, the foot’s CoP is positioned more medially, and the H-GRF (heel vGRF) increases sharply. On uneven/slippery ground, slipping and falling may occur during the push (pre-swing) and loading (heel contact) phases. These findings may provide valuable insights for rehabilitation professionals dealing with populations at risk of falls and lacking gait stability.

## Figures and Tables

**Table 1:** Multiple linear regression results for the left CoP, left GRF, and right GRF models.

Dependent Variables (Lt. Push Phase)		Independent Variables (Non-Multicollinearity)	Standardized Regression Coefficient (β)	Coefficient of Determination (R^2^)	*p* Value
Step-aside movement	CoPx	PL	0.465	0.216	<0.001
	CoPy	PL	0.549	0.355	<0.001
		TA	−0.215		0.011
	F-GRF	PL	0.635	0.890	<0.001
		CoPx	0.423		<0.001
		TA	−0.168		<0.001
Straight walking	CoPx	TA	−0.604	0.364	<0.001
	CoPy	TA	−0.311	0.243	0.002
		PL	0.288		0.003
	F-GRF	CoPy	0.291	0.760	<0.001
		SOL	0.715		<0.001
**Dependent Variables** **(Rt. Loading Phase)**		**Independent Variables (Non-Multicollinearity)**	**Standardized Regression Coefficient (β)**	**Coefficient of Determination (R^2^)**	***p* Value**
Step-aside movement	H-GRF	CoPy	−0.450	0.202	0.002
Straight walking	H-GRF	CoPx	1.467	0.898	<0.001
		CoPy	−1.033		<0.001
		PL	0.208		<0.001

All β values in the table are statistically significant (*p* < 0.05). Independent variables with multicollinearity were excluded from the analysis. The independent variables were arranged in ascending order of condition index (<15). TA, tibialis anterior; PL, peroneus longus; SOL, soleus; CoP, center of pressure; GRF, ground reaction force; F-GRF, forefoot ground reaction force; H-GRF, heel ground reaction force.

## Data Availability

The data are available in a public, open-access repository. Repository name: Open Science Framework database. Registration DOI: https://doi.org/10.17605/OSF.IO/XVNAQ. Internet Archive link: https://archive.org/details/osf-registrations-xvnaq-v1, accessed on 8 August 2023.

## References

[1] Xie L., Cho S. (2023). Ankle strategies for step-aside movement during quiet standing. PLoS ONE.

[2] Courtine G., Schieppati M. (2003). Human walking along a curved path. II. Gait features and EMG patterns. Eur. J. Neurosci..

[3] Haefeli J., Vögeli S., Michel J., Dietz V. (2011). Preparation and performance of obstacle steps: Interaction between brain and spinal neuronal activity. Eur. J. Neurosci..

[4] Grin L., Frank J., Allum J.H. (2007). The effect of voluntary arm abduction on balance recovery following multidirectional stance perturbations. Exp. Brain Res..

[5] Michel J., Van Hedel H., Dietz V. (2008). Obstacle stepping involves spinal anticipatory activity associated with quadrupedal limb coordination. Eur. J. Neurosci..

[6] Hof A., Duysens J. (2018). Responses of human ankle muscles to mediolateral balance perturbations during walking. Hum. Mov. Sci..

[7] Hof A.L., van Bockel R.M., Schoppen T., Postema K. (2007). Control of lateral balance in walking: Experimental findings in normal subjects and above-knee amputees. Gait Posture.

[8] Van Leeuwen A., Van Dieën J., Daffertshofer A., Bruijn S. (2021). Ankle muscles drive mediolateral center of pressure control to ensure stable steady state gait. Sci. Rep..

[9] Hase K., Stein R. (1999). Turning strategies during human walking. J. Neurophysiol..

[10] Wikstrom E.A., Hass C.J. (2012). Gait termination strategies differ between those with and without ankle instability. Clin. Biomech..

[11] Hoogkamer W., Potocanac Z., Duysens J. (2015). Quick foot placement adjustments during gait: Direction matters. Exp. Brain Res..

[12] Kim K.-J., Uchiyama E., Kitaoka H.B., An K.-N. (2003). An in vitro study of individual ankle muscle actions on the center of pressure. Gait Posture.

[13] Novacheck T.F. (1998). The biomechanics of running. Gait Posture.

[14] Rose J., Gamble J.G. (1994). Human Walking.

[15] Tretriluxana J., Nanbancha A., Sinsurin K., Limroongreungrat W., Wang H.-K. (2021). Neuromuscular control of the ankle during pre-landing in athletes with chronic ankle instability: Insights from statistical parametric mapping and muscle co-contraction analysis. Phys. Ther. Sport.

[16] Gibbons C.T., Amazeen P.G., Likens A.D. (2019). Effects of foot placement on postural sway in the anteroposterior and mediolateral directions. Mot. Control..

[17] Insperger T., Milton J. (2021). Delay and Uncertainty in Human Balancing Tasks.

[18] Franklin D.W., Osu R., Burdet E., Kawato M., Milner T.E. (2003). Adaptation to stable and unstable dynamics achieved by combined impedance control and inverse dynamics model. J. Neurophysiol..

[19] Heald J.B., Franklin D.W., Wolpert D.M. (2018). Increasing muscle co-contraction speeds up internal model acquisition during dynamic motor learning. Sci. Rep..

[20] Babadi S., Vahdat S., Milner T.E. (2021). Neural Substrates of Muscle Co-contraction during Dynamic Motor Adaptation. J. Neurosci..

[21] Hermens H.J., Freriks B., Merletti R., Stegeman D., Blok J., Rau G., Disselhorst-Klug C., Hägg G. (1999). European recommendations for surface electromyography. Roessingh Res. Dev..

[22] Rankin B.L., Buffo S.K., Dean J.C. (2014). A neuromechanical strategy for mediolateral foot placement in walking humans. J. Neurophysiol..

[23] Noraxon Ultium Insole Quick Start Guide. https://www.noraxon.com/noraxon-download/ultium-insoles-quick-start-guide.

[24] Orpyx Medical Technologies (Calgary, Canada) (2017). Orpyx LogR: Validation of Plantar Pressure Measurement Performance.

[25] Pataky T.C. (2012). One-dimensional statistical parametric mapping in Python. Comput. Methods Biomech. Biomed. Eng..

[26] Pataky T.C., Robinson M.A., Vanrenterghem J. (2016). Region-of-interest analyses of one-dimensional biomechanical trajectories: Bridging 0D and 1D theory, augmenting statistical power. PeerJ.

[27] Pataky T.C. (2010). Generalized n-dimensional biomechanical field analysis using statistical parametric mapping. J. Biomech..

[28] Cao J., Worsley K.J. (1999). The detection of local shape changes via the geometry of Hotelling’s *T*^2^ fields. Ann. Statist..

[29] Pataky T.C., Robinson M.A., Vanrenterghem J. (2013). Vector field statistical analysis of kinematic and force trajectories. J. Biomech..

[30] Adler R.J., Taylor J.E. (2007). Random Fields and Geometry.

[31] Pataky T.C. (2016). RFT1D: Smooth one-dimensional random field upcrossing probabilities in Python. J. Stat. Softw..

[32] Pataky T.C., Vanrenterghem J., Robinson M.A. (2016). The probability of false positives in zero-dimensional analyses of one-dimensional kinematic, force and EMG trajectories. J. Biomech..

[33] Yiou E., Caderby T., Delafontaine A., Fourcade P., Honeine J.-L. (2017). Balance control during gait initiation: State-of-the-art and research perspectives. World J. Orthop..

[34] Bavdek R., Zdolšek A., Strojnik V., Dolenec A. (2018). Peroneal muscle activity during different types of walking. J. Foot Ankle Res..

[35] Martin P.E., Marsh A.P. (1992). Step length and frequency effects on ground reaction forces during walking. J. Biomech..

